# A Murine Model to Study Epilepsy and SUDEP Induced by Malaria Infection

**DOI:** 10.1038/srep43652

**Published:** 2017-03-08

**Authors:** Paddy Ssentongo, Anna E. Robuccio, Godfrey Thuku, Derek G. Sim, Ali Nabi, Fatemeh Bahari, Balaji Shanmugasundaram, Myles W. Billard, Andrew Geronimo, Kurt W. Short, Patrick J. Drew, Jennifer Baccon, Steven L. Weinstein, Frank G. Gilliam, José A. Stoute, Vernon M. Chinchilli, Andrew F. Read, Bruce J. Gluckman, Steven J. Schiff

**Affiliations:** 1 Center for Neural Engineering, Penn State University, University Park, Pennsylvania 16802, USA; 2 Department of Engineering Science and Mechanics, Penn State University, University Park, Pennsylvania 16802, USA; 3 Department of Public Health Sciences, Penn State College of Medicine, Hershey, Pennsylvania 17033, USA; 4 Center for Infectious Disease Dynamics, Penn State University, University Park, Pennsylvania 16802, USA; 5 Departments of Biology and Entomology, Penn State University, University Park, Pennsylvania 16802, USA; 6 Department of Neurosurgery, Penn State College of Medicine, Hershey, Pennsylvania 17033, USA; 7 Department of Neurology, Penn State College of Medicine, Hershey, Hershey, Pennsylvania 17033, USA; 8 Department of Pathology, Penn State College of Medicine, Hershey, Hershey, Pennsylvania 17033, USA; 9 Department of Neurology, Children’s National Medical Center, George Washington University, Washington, DC 20010, USA; 10 Department of Medicine, Penn State University College of Medicine, Hershey, Pennsylvania 17033, USA; 11 Department of Bioengineering, Penn State University, University Park, Hershey, Pennsylvania, 16803, USA; 12 Department of Physics, Penn State University, University Park, Pennsylvania, 16803, USA

## Abstract

One of the largest single sources of epilepsy in the world is produced as a neurological sequela in survivors of cerebral malaria. Nevertheless, the pathophysiological mechanisms of such epileptogenesis remain unknown and no adjunctive therapy during cerebral malaria has been shown to reduce the rate of subsequent epilepsy. There is no existing animal model of postmalarial epilepsy. In this technical report we demonstrate the first such animal models. These models were created from multiple mouse and parasite strain combinations, so that the epilepsy observed retained universality with respect to genetic background. We also discovered spontaneous sudden unexpected death in epilepsy (SUDEP) in two of our strain combinations. These models offer a platform to enable new preclinical research into mechanisms and prevention of epilepsy and SUDEP.

Of the greater than 200 million people who contract malaria each year^1^, cerebral malaria (CM) affects more than 3 million^2^. CM typically affects children under 5 years of age, and carries high mortality rates even with the availability of antimalarial treatment^2^. The prevalence of epilepsy (proportion of the population with epilepsy) in malaria endemic countries is 2–6 times than that of the industrialized counties^3^. Epidemiological studies report that rates of epilepsy as a sequela in survivors of CM range from 5–17%^2^,^4^,^5^. CM is therefore one of the largest single sources of epilepsy on the planet. Nevertheless, the pathophysiological mechanisms of epileptogenesis remain unknown and no adjunctive therapy during CM has been shown to reduce the rate of subsequent epilepsy.

Epilepsy leads to a 2.6-fold increased risk of premature death in industrialized countries^6^, and a rate as high as 6% in developing countries^3^. One source of this increased mortality is death associated with seizures, including the syndrome of sudden unexplained death from epilepsy (SUDEP). SUDEP incidence rates range from 0.1 to 9.0 per 1000 person-years depending on the types of epilepsy^7^. SUDEP is the leading cause of premature death among patients who are pharmacologically resistant to antiepileptic medication^8^. The standardized mortality rate for SUDEP in young epileptics (20–40 years) is 24-times the rate of the general population^9^.

In this technical report, we demonstrate the first animal models of postmalarial epilepsy. These models were created from multiple mouse and parasite strain combinations, so that the epilepsy observed retained some universality with respect to genetic background. To establish epilepsy, we implemented chronic continuous (i.e. twenty-four hours, seven days per week) video/EEG monitoring, and utilized a clinically derived definition that required observation of at least two spontaneous (i.e. not associated with the acute infection, its recovery, nor externally provoked) seizures. We also discovered spontaneous SUDEP in two of our strain combinations. These models offer a platform to enable new preclinical research into mechanisms and prevention of epilepsy and SUDEP.

## Results

We examined 6 combinations of mouse and parasite strains by crossing Swiss-Webster (SW), C57BL/6, and CBA mice with Plasmodium berghei ANKA (PbANKA) and Plasmodium berghei NK65 (PbNK65) parasite strains. Mice were infected at young age (P23) because older mice have less tendency to develop CM during malaria^10^. Infection was accomplished with homologous donor blood from infected animals. Littermate controls for each mouse strain were inoculated with uninfected homologous donor blood.

**Figure 1:**
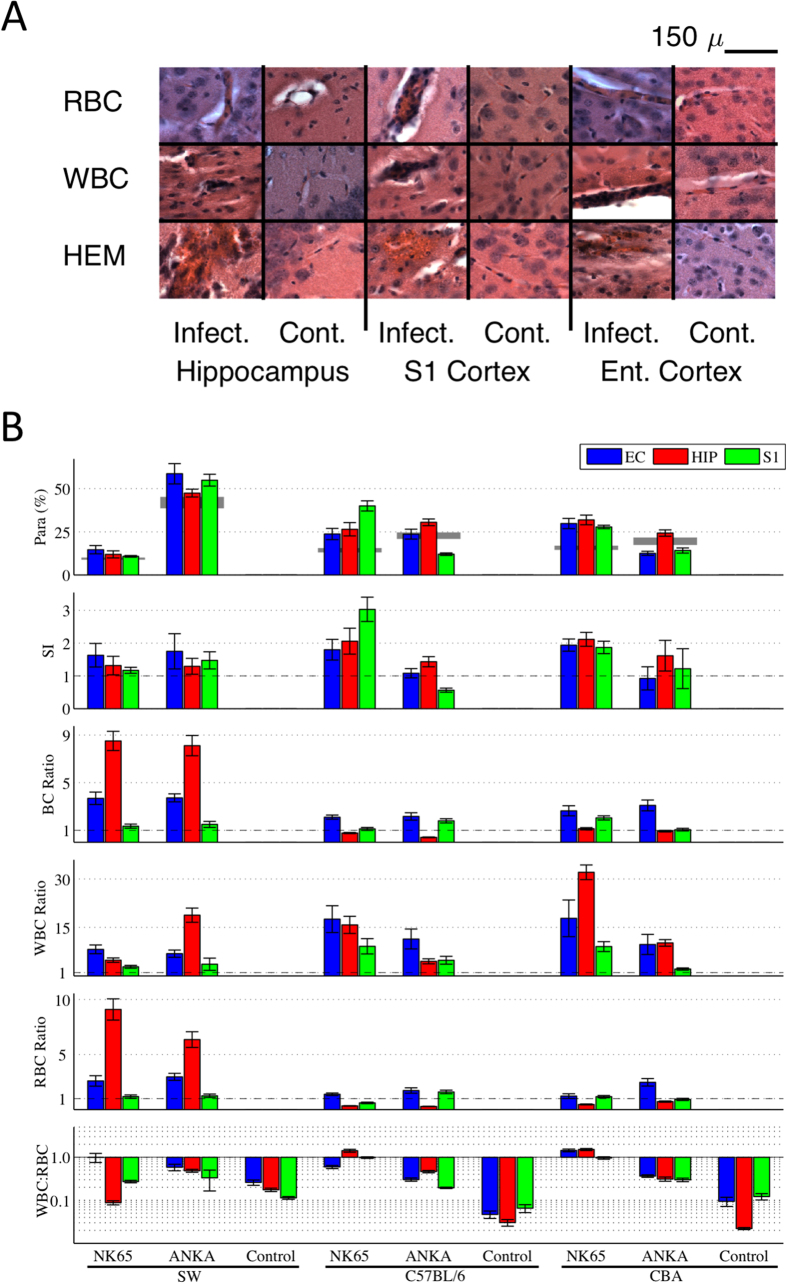
Histological characterization of acute cerebral malaria. (**A)** Examples of the appearance of sequestration of red blood cells (RBC) and white blood cells (WBC), as well as hemorrhages (HEM) in hippocampus, primary somatosensory (S1) and entorhinal cortex (Ent.), in infected (Infect.) versus control uninfected animals (Cont.). In sequestration, the blood cells are accumulating within blood vessels, whereas in hemorrhage the blood cells are extravascular. No cerebral vessel congestion or hemorrhage was observed in control mice. Magnification 100X, scale bar 150 μm. (**B)** Quantitative brain histological characteristics from different mouse-strain and parasite combinations from animals sacrificed at peak CM infection. Each block represents mean and standard error of the mean of values averaged over cohorts of 10 animals. The top row is the fraction of RBCs infected with parasite within the brain (parasitemia, Para, in %), with the peripheral parasitemia level indicated with gray. The second row is the ratio of brain to peripheral parasitemia known in human CM pathology as the sequestration index (SI). SI ratios greater than 1 indicate trapping of iRBCs within the brain, a hallmark of human CM. Note that most strain combinations have evidence of sequestration, but that it varies by brain region. The blood cell densities in rows 3–5 are all normalized by region and strain-specific control values, so control values appear at 1. The third row details the total blood cell (BC) density, a composite of WBC and RBC densities in rows four and five, normalized by the individual mouse strain control values. In the WBC/RBC ratio (plotted on log scale in the sixth row) we find unusually elevated ratios consistently equal or greater than 1 for strain/parasite combinations C57BL/6-PbNK65 and CBA-PbNK65. Because such high densities have not been reported in human histology from CM, these two strain combinations were eliminated from further study. Note that all control WBC/RBC ratios were less than 1. For each mouse strain, shipments of 30 animals were distributed evenly and randomly among each of 3 inoculation groups (PbANKA, PbNK65, Control) so that controls included littermates of infected animals. Bars indicate ± 1 s.e.m.

We first studied brain physiology of animals at the peak of infection. For each strain combination, we examined the brain histology from the acute infectious stage (Fig. 1A) to establish the similarity with human disease. We quantified region specific densities within the brain (Fig. 1B) of total blood cells (BC), total red blood cells (RBC) and infected RBC (iRBC), white blood cells (WBC), the ratio WBC/RBC, and the sequestration index (SI). Each of the raw blood cell densities are normalized by the region specific densities in the uninfected littermate controls. The SI^12^ is the fraction of RBC infected with parasite within the brain divided by the fraction of infected RBC within the peripheral blood, and has been considered a hallmark of the pathophysiology of human CM^13^.

There were three aspects of the descriptive acute histology that we point out. First, in 2 strain combinations, C57BL/6-PbNK65 and CBA-PbNK65, the WBC/RBC ratios within the brain were consistently greater than 1 across multiple brain regions during acute infection (Fig. 1B, statistical analysis in Supplemental material). Because such ratios are inconsistent with human histology^13^,^14^,^15^, we excluded these 2 strain combinations from further study. Second, for SW-PbNK65 and SW-PbANKA, the BC densities could be 8–10 fold higher than controls. We separately quantified the fraction of RBC density within the brain due to hemorrhage, and found that it constituted less than 5% of these BC densities. Because the normal mouse hematocrit is 40% or higher, an increase in within-vasculature blood density can only account for as much as a 2.5 times increase. Therefore this increased RBC density indicates an increase in vascular volume. The third observation is that there are a variety of sequestration or microvascular trapping-type effects observed in the RBC, WBC and BC densities that are not consistently reflected by the SI metric, consistent with other recent findings^16^,^17^.

We next took cohorts of the 4 strain combinations selected for further study, and treated them for malaria. Littermate control animals inoculated with uninfected donor blood were treated identically.

Mice infected with malaria that experience CM undergo a progression of behavioral changes that are indicators of disease and possible neurological deficits. To track the progression non-invasively, we used a behavioral scale (BS) modified from Caroll, *et al*.^11^: (BS0) Normal activity; (BS1) Poor grooming including observation of ruffled hair; (BS2) Slow movement, including hunched body posture; (BS3) Tendency to roll over on stimulation, ataxia, evidence of hemi- or para-plegia, ~10% body weight loss; (BS4) Comatose, convulsions, >20% body weight loss. For all the mouse/strain combinations studied, BS1 (ruffled hair) was observed by day 3, and BS4 between day 5 and 7. If not treated on the day they reach BS4, we observe ~80% mortality rate. Pilot studies were used for each combination studied to establish this time point and therefore identify both the peak infection time and treatment time points for the acute and chronic studies reported here.

**Figure 2:**
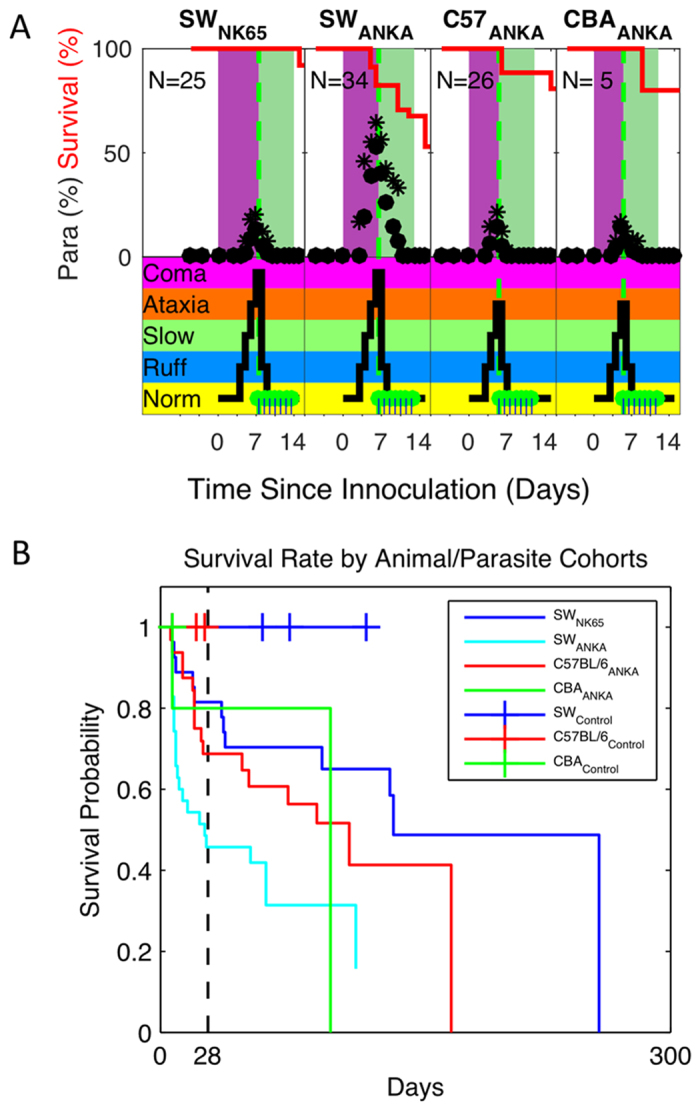
Parasitemia, Behavioral Signs and Survival curves from chronic monitoring cohorts. (**A**) Shown in the upper panels are the peripheral parasitemia levels (average •, maxima *) for each mouse/strain combination, along with the survival probability (red line) through the infection (purple shading) and treatment (green shading) phases. Shown in the lower panel on the same time frame are the behavioral scale (BS) values, adapted from Caroll, *et al*.^11^. The green dots mark individual treatment times. BS values include: **Norm** – Normal behavior; **Ruff** - Poor grooming including observation of ruffled hair; **Slow** - Slow movement, including hunched body posture; **Ataxia** - Tendency to roll over on stimulation, ataxia, evidence of hemi- or para-plegia, ~10% body weight loss; **Coma** - Comatose, convulsions, >20% body weight loss. Note that within 1 day of treatment, infected animals’ parasitemia drops by approximately half and their behavior returns nearly to normal. (**B**) Shown are Kaplan-Meir curves for survival by mice with CM by animal-parasite strain combinations for all animals inoculated for chronic monitoring. Those sacrificed according to protocol, malarial recrudescence, or associated with surgery were censored. Note that the mouse strain SW demonstrated the shortest survival rates when infected with PbANKA (log-rank test p < 0.05), in contrast to the longest survival rates for SW infected with PbNK65.

The time course of both the parasitemia levels, the associated behavioral signs of the infection, and the survival probability for all of the infected cohorts are shown in Fig. 2A through the first 20 days post-inoculation. Note that for all strain/parasite combinations studied, the animal’s overt appearance deviates from normal only in terms of grooming, and their parasitemia levels are halved within 2 days of treatment. The survival of the treated strain combinations varied despite treatment with artesunate during infection, with early mortality taking its greatest toll on SW-PbANKA (log-rank test p < 0.05, Fig. 2A,B, Table 1).

The 4 strain combinations studied for postmalarial epilepsy were implanted after day 17 postinfection with a combination of epidural and depth electrodes, along with electromyography (EMG) electrodes, and chronically recorded 24/7 with both video and continuous intracranial electroencephalography (iEEG). Fifteen (15) animals also had a precordial lead implanted.

**Figure 3:**
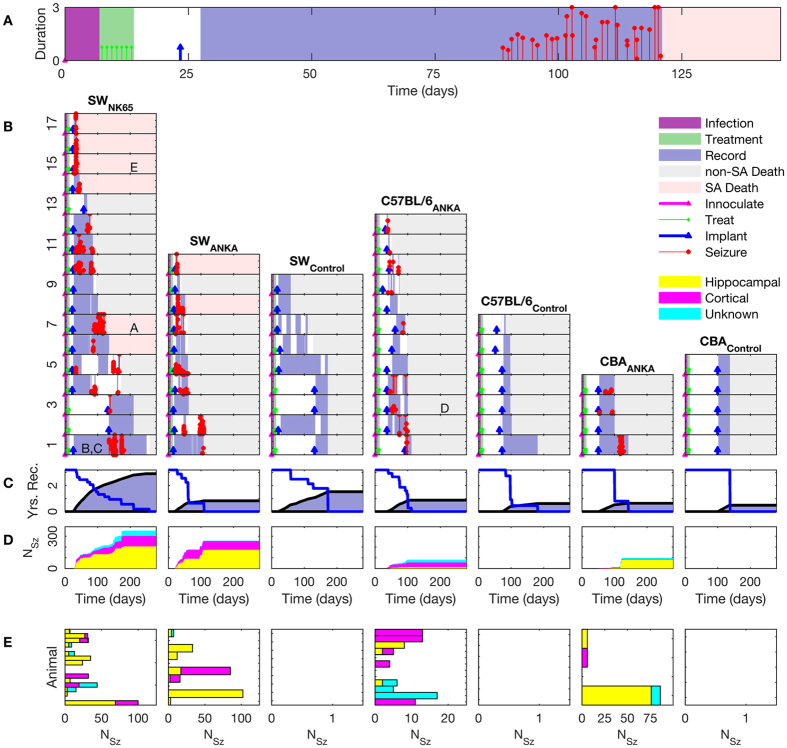
Summary time courses for each cohort and animal. (**A)** Typical time course of an experiment from inoculation through to seizure associated (SA) death. (**B**) Time courses of all recorded animals, with columns for experimental strain combinations and control cohorts, and row for individual animals. The time courses begin with inoculation (magenta triangle) at day 0, and the period of infection is marked in purple. Treatment is indicated with green markers, the time of electrode implantation indicated with blue markers, and the periods of recording marked with blue background. The time of seizure occurrence over 10 s is indicated by red markers, with height representing duration in minutes. Time of death is indicated by the transition to grey (from investigator sacrifice, non-SA death) or pink (SA death) shading. Spontaneous deaths from the SA-death cohort forms the group further analyzed for possible SUDEP. Lettering within (**A–E**) correlate subjects with the seizure example panels in Fig. 4. (**C**) Cumulative time of recordings in grey and uncensored survival curves for each strain combination. Total recording time represented is 2751 days. (**D)** Cumulative number of seizures for each strain combination, with seizure origins indicated as hippocampal, cortical, and unknown. The cumulative number of seizures (>10 s long) marked is 786, with none observed from control animals. (**E)** Total number of seizures by category for each animal. Multiple seizure types are frequently seen in individual animals in the postmalarial epilepsy cohorts, while no seizures were recorded in any of the control animal cohorts.

To be included in recording analysis, animals needed to survive and be recorded for at least 2 days. To be considered epileptic, the animal was required to have survived at least 26 days after inoculation, had at least 2 greater than 10 s long seizures recorded and no recrudescence of malaria. The time courses of all chronic experiments are summarized in Fig. 3. We identified 2 animals with recrudenscence after day 21, which were included in our mortality figures (as a non-seizure related mortality, Table 1), but excluded from Fig. 3.

In Fig. 3A, we detail the experimental time course for one mouse, illustrating the time of inoculation (magenta triangle) at day 0, and the period of infection shaded in purple. Treatment with artesunate is indicated with green markers, and takes place during the shaded green time interval. Electrode implantation is indicated after day 21 (blue triangle). The period of continuous recording is indicated in light blue shading. Note that long latencies were observed in some animals before seizures were observed (red markers). In the example in Fig. 3A, 89 days elapsed after inoculation prior to the first seizure, after 61 days of continuous video and iEEG monitoring. The epilepsy diagnosis required 2 or more spontaneous seizures during this post-infectious period.

In Fig. 3, death that occurred spontaneously and was associated in time with the presence of seizures is indicated with pink shading (as opposed to planned sacrifice indicated with grey shading). It is within these seizure associated deaths that some cases met our criteria for SUDEP – death within 2 hours of a seizure in an animal otherwise stable prior to the seizure.

In Fig. 3B, we detail the time courses of all animals sorted by strain combination and controls. The orderings of the experiments are done in descending order with respect to length of survival within each cohort (1 is the longest survival). Note the substantial latencies to the occurrence of the first observed seizure among animals classified as epileptic, with median latencies per cohort from 30–72 days, and ranges from 22–139 days (Table 1).

Latencies reported are upper bounds on time to first clinical seizure with duration longer than 10 s, and are limited by the onset of our recordings. As illustrated in Fig. 3B, recordings on most animals initiated close to day 27 post inoculation, and proceeded continuously from there. Implantation and recording was delayed in a few animals (eg. SW-PBNK65 numbers 2, 3, and 13) to allow for recording later in their lives. For these animals the reported latencies are consistent with the range of latencies observed in animals from the same cohort whose recordings proceeded continuously from day 27.

The cumulative recording time in years is shown in Fig. 3C, along with the cumulative mortality. Note columns in both Fig. 3B and C share the time (horizontal) axes. These data represent 2751 mouse-days of total recording (1981 days of recordings in N = 43 postinfectious animals, and 770 days of recording in N = 21 control animals). These represent substantially continuous recordings, with occasional breaks due to computer failure or accommodate computer maintenance. Recordings were also interrupted on longer time scales for rotation between animals to accommodate the availability of recording rigs, but typically only after multi-week recording sessions. This on/off duty cycle in recording is marked in Fig. 3B with the breaks in the blue shading.

The cumulative number of seizures (786), based upon origin (subcortical hippocampal, cortical, or unknown), are shown by animal in Fig. 3D. No seizures were observed from any of the control mice, which were inoculated with uninfected homologous donor blood, and received the same pharmacological treatments and electrode implantations.

The distribution of seizures by animal, in order of the experiments in Fig. 3B, and the origin of the seizures, are shown in Fig. 3E.

Of 90 infected animals, there were 32 that became epileptic among the 43 that survived treatment and implantation (Table 1). Epilepsy rates ranged from 21–60% of the initial cohort sizes, or 70–76% of those surviving to implant (Table 1). The difference between these reflects early mortality. Spontaneous mortality in epileptic animals ranged from 33–54% during recordings. Three of these deaths met our strict criteria for SUDEP (2 in SW-PbNK65, 15%; 1 in SW-PbANKA, 14%).

Epilepsy was defined based upon observation of seizures with durations longer than 10 s and clear behavioral correlates. This classification provided a conservative interpretation measure of the development of epilepsy as a sequela of CM. Prior to the observation of the first clinical seizure, a progression of different abnormal rhythms were observed in the iEEG, starting from first isolated epileptic spikes to bursts to trains of spikes or spike-wave discharges. It was difficult to find behavioral correlates in the video for these brief events. A detailed analysis of their evolution will be presented elsewhere.

**Figure 4:**
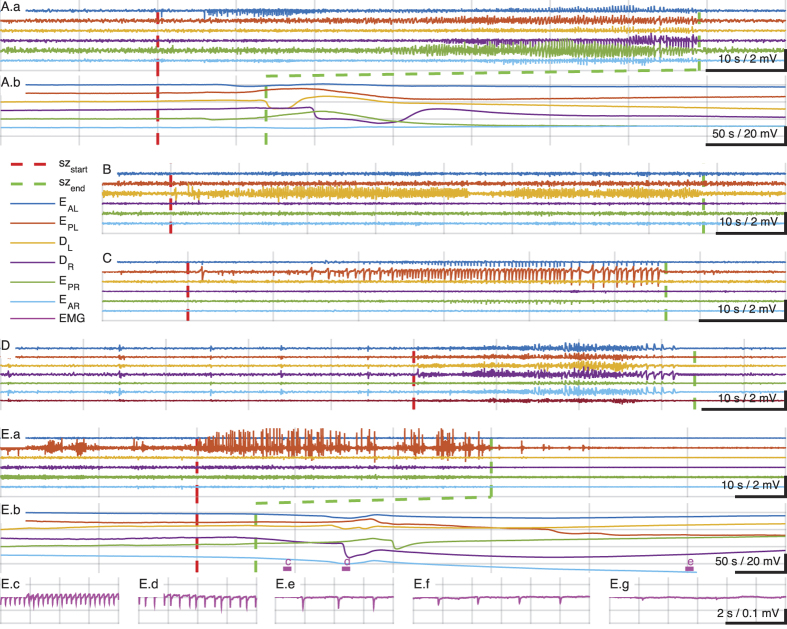
Examples of seizure types and SUDEP. Seizures were classified using the 2010 International League against Epilepsy seizure classification system^18^ depending on the mode of onset and semiology using video-EEG recordings. (**A.a**) Seizure of cortical onset with secondary generalization. In compressed time-scale below, (**A.b**), note direct current (DC) potential changes in cortical and hippocampal electrodes consistent with propagating spreading depression (SD) following seizure termination (vertical broken green line). In (**B**) is shown a focal hippocampal seizure, and in **C** a focal cortical seizure, both from the same animal. (**D**) Illustrates an example of a primary generalized seizure preceded by a series of pre-ictal generalized spikes. In (**E**) is shown an example of a sudden death during seizure. The cortical focal seizure shown in (**E.a**) is punctuated by the animal becoming behaviorally quiet, and is followed by propagating depolarizations consistent with SD, shown in (**E.b**). Following the seizure, the muscle activity is quiet enough to reveal the EKG reflected in the EMG lead which demonstrates progressive bradycardia leading to asystole shown in (**E.c**–**E.g**). EEG montage: EAL, EEG anterior left (frontal); EPL, EEG posterior left (somatosensory); DL, depth hippocampus left; DR, depth hippocampus right; EAR, EEG anterior right, EPR, EEG posterior right. Filter settings for traces shown: Seizure traces bandpass 1–50 Hz; SD traces low-pass below 1 Hz; EMG/EKG traces 0.1–55 Hz.

Examples of the iEEG from individual seizures are shown detail in Fig. 4. The seizure onsets are indicated by vertical dashed red lines, and the seizure offsets by vertical dashed green lines. A wide variety of seizure types and origins were identified including focal with secondary generalization (Fig. 4A), focal subcortical (Fig. 4B), focal cortical (Fig. 4C), and primary generalized (Fig. 4D, note the preictal iEEG generalized spikes and myoclonic jerks, also seen on video and EMG not shown). In Fig. 4E, we show a focal cortical seizure leading to SUDEP. Note the EKG reflected in the EMG electrode lead, demonstrating progressive bradycardia in Ec through Ef, leading to asystole by Eg (Ef and Eg are out of range of the trace Eb). Our custom recording system and electrodes (see Methods) permitted low frequency and direct current (DC) recordings to accompany these iEEG measurements, and we illustrate patterns of propagating depolarizations similar to spreading depression^19^ following seizure activity in Fig. 4A.b and E.b (at a 5x compressed time scale).

**Figure 5:**
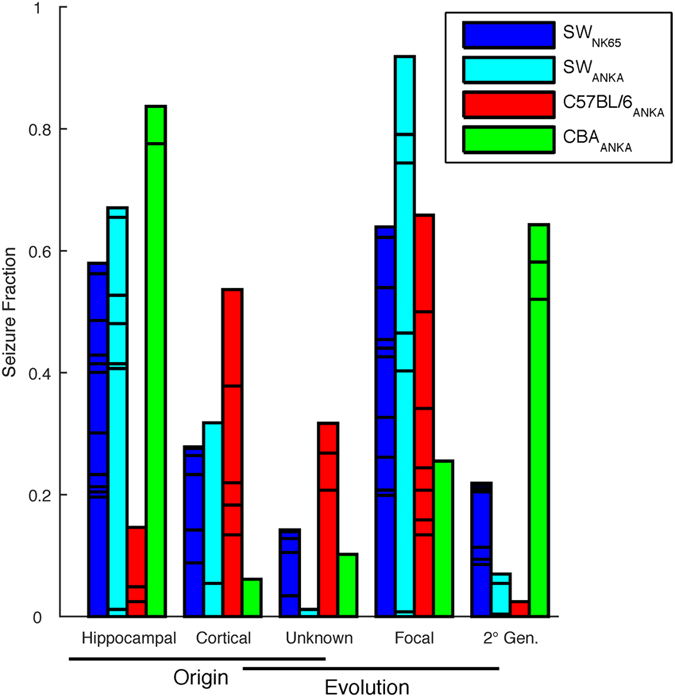
Seizure Origin and Evolution. Seizure characterizations by origin within the brain and evolution of focal versus generalized seizure. Subdivisions within bars represent different animals. Note that each color represents a strain combination, and the individual counts are normalized by total number of seizures, so within each color-coded cohort the first 3 columns, and the last 3 columns, of a given color add to 1.

A summary of the fraction of seizures that originate in cortex versus subcortical regions, or were focal versus generalized, is summarized by strain combination in Fig. 5. There were a wide variety of origins and seizure evolution patterns observed, consistent with the widespread and heterogeneous effects of CM on the brain.

## Discussion

We here report the first animal models of postmalarial epilepsy. In previous work, seizures have been observed during the acute infectious stage of CM^20^,^21^,^22^, but spontaneous recurrent seizures following infection have not been observed^22^. We observed epilepsy as a sequela of CM across genetic background of host and parasite combinations for both outbred stock (SW) and inbred animal strains (CBA and C57BL/6). Our goal was to develop a model that was robust across background genetic heterogeneity of host and parasite. We sought to achieve a degree of universality in that inferences derived from experiments in such heterogeneous models would not be restricted to a particular genetic background, and might better enable future human clinical trials of adjunctive therapy during CM.

CM is a syndrome that has a predilection for human children, and we targeted juvenile mice (P23) in our experiments. We were impressed by the long latency required to observe epilepsy (more than 2 spontaneous convulsive seizures over 10 s) in such animals, waiting as long as 139 days to observe epilepsy following infection (Table 1). In human studies, long latencies from recovery from CM to the onset of seizures are also seen, with recent documentation of a median of 309 days (range 111–524 days)^4^.

We quite unexpectedly encountered SUDEP in several animals. We gave strict criteria to the animals we labeled as SUDEP as being stable prior to a final seizure, and then expiring within 2 hours. Nevertheless, we have observed gradual declines in other animals following repeated seizures, which although not consistent with typical SUDEP definitions^23^,^24^, may indeed be consistent with recent physiological demonstrations of the effect of spreading depression on brain function in other animal models of SUDEP^25^. To our knowledge, no chronic video-EEG recordings of spontaneous SUDEP in animal models has been previously accomplished, and our ability to record chronic high quality DC biopotentials along with our iEEG offers a technical platform to further investigate SUDEP pathophysiology.

Although there are many prior animal models of epilepsy^26^, there are few with postinfectious spontaneous seizures (such as the Theiler’s viral model^27^) that mimic human epilepsy as a sequela of infection. Similarly, although there are genetic mutant models of SUDEP, we are unaware of chronic spontaneous SUDEP in an animal model whose epilepsy reflects a common human epilepsy.

There are a number of preliminary recommendations we might make to investigators seeking to select strain combinations among this suite of models. The highest acute mortality during infection and early recover was seen in the SW-PbANKA, which led to the lowest yield of epileptic animals (Table 1). Of the animals who survived CM to implant, three quarters of the animals from each strain combination became epileptic. Most of the seizure-associated deaths were observed in the SW animals, including all of the animals which met our criteria for SUDEP.

Postmalarial epilepsy may be one of the world’s most prominent sources of epilepsy, but until now, there has been no animal model to enable a detailed examination of the pathophysiology, and no way experimentally to develop adjunctive therapies to prevent such epilepsy. Postmalarial epilepsy is one that can be prevented if we can use animal models of cerebral malaria to help develop more effective adjunctive antiepileptogenesis therapy.

## Methods

All protocols and procedures were approved by the Animal Care Committee of the Pennsylvania State University, University Park, and all experiments were performed in accordance with relevant guidelines and regulations.

### Overview

Juvenile mice infected with cerebral malaria inducing parasites at day P23, along with littermate controls, were studied under 2 experimental protocols: histological analysis during the infectious phase to assess commonalities with the human cerebral malaria, and long-term monitoring of treated animals for the development of epilepsy.

Commonalities to both protocols include the mice and parasite handling, disease assessment and blood parasite (parasitemia) monitoring. Animals monitored long term were first treated for malaria, allowed to recover and then implanted with electrodes.

### Mice

Swiss Webster (SW), C57BL/6 (Charles Rivers Laboratory) and CBA/CaJ (CBA, Jackson Laboratory) male mice were housed in a temperature-controlled room with a 12/12 hour dark/light cycle with *ad libitum* access to water and food. All studies were initiated in mice age 3 weeks.

### Parasites, Disease Assessment, Parasitemia Monitoring, Treatment

Red blood cells infected with Plasmodium berghei ANKA (PbANKA) or Plasmodium berghei NK65 (PbNK65) were used to infect SW, C57BL/6 and CBA mice. Donor animals were infected from frozen stocks of parasite, and blood drawn on day 7 for inoculation into homologous experimental animals. Mice were infected by intraperitoneal inoculation of 10^6^ infected red blood cells (iRBC). Control mice were inoculated with 10^6^ non-infected red blood cells (RBC) from uninfected homologous donor animals.

Mice for long-term recordings were rescued with Artesunate 64 mg/kg twice a day for 7 days starting on day 5 (C57BL/6-PbANKA or CBA-PbANKA), day 6 (SW-PbANKA) and day 7 (SW-PbNK65) post-infection. These treatment initiation times were 1.5–2 days prior to the typical day of death of each untreated strain combination and were chosen through a pilot study to optimize between significant clinical signs of ECM and survival through treatment. Our conjecture was that a substantial impact upon the brain from CM would be required to predispose the animal to future epilepsy, but this created a narrow window remaining within which antimalarial rescue would still be effective.

During infection and treatment phases, all mice were monitored three times daily for clinical symptoms of experimental cerebral malaria (ECM) including tendency to roll over on stimulation, hemi- or paraplegia, head deviation, ataxia, convulsions and coma.

Parasitemia was determined by Giemsa staining of blood drawn by tail snip followed by microscopic quantification. These results are expressed as percentage of infected red blood cells.

Parasitemia was monitored daily during the infectious and treatment phases. For treated animals, after parasitemia levels dropped to zero and treatment ended, parasitemia monitoring rate dropped to once per week unless indicated either by moribund presentation, or indicated by relapse of the animal or one of its littermates.

Animals were received from vendors in packages of 10 containing littermates, and divided between experimental and controls groups such that each infected cohort had at least 2 littermate controls. Control animals received identical tail snips for parasitemia monitoring and artesunate treatment as infected animals.

### Infectious Phase Experiments

In order to study the acute brain insult from the infection, cohorts of 10 animals for each mouse-strain and parasite combination along with littermate controls were inoculated with infected/non-infected blood at P23, their parasitemia monitored daily, sacrificed, and their brain histology quantified using stereological methods^28^,^29^ for signs of sequestration and damage. Day of sacrifice was targeted to maximize neurological correlates to human CM while minimizing mortality – on day 7 post inoculation for the SW parasite or control combinations, and on day 6 for the C57BL/6 and CBA combinations.

#### Brain fixation and sectioning

Mice were deeply anesthetized with inhalation isoflurane and decapitated. To avoid tissue artifact due to handling of the fresh brain, intact skulls were submerged in 4% paraformaldehyde for 72 hours, then the brain were removed and placed in cryoprotectant (4% paraformaldehyde and 30% sucrose) for at least another week prior to sectioning. Brains were paraffin embedded. Serial 25 μm coronal sections were collected from Bregma −0.94 mm to Bregma −3.88 mm. Every 25th brain section was stained with hematoxylin and eosin (H&E) starting from a random initial point in the series for analysis.

#### Stereological Methodology

The iRBC, white blood cells (WBC) and RBC were counted in the dentate gyrus (DG), Cornu Ammonis (CA) CA1 and CA3 of the hippocampus, primary somatosensory (S1) and entorhinal cortex (EC) regions (Fig. 1A) using a systematic uniform random sampling principle^28^,^29^.

**Figure 6:**
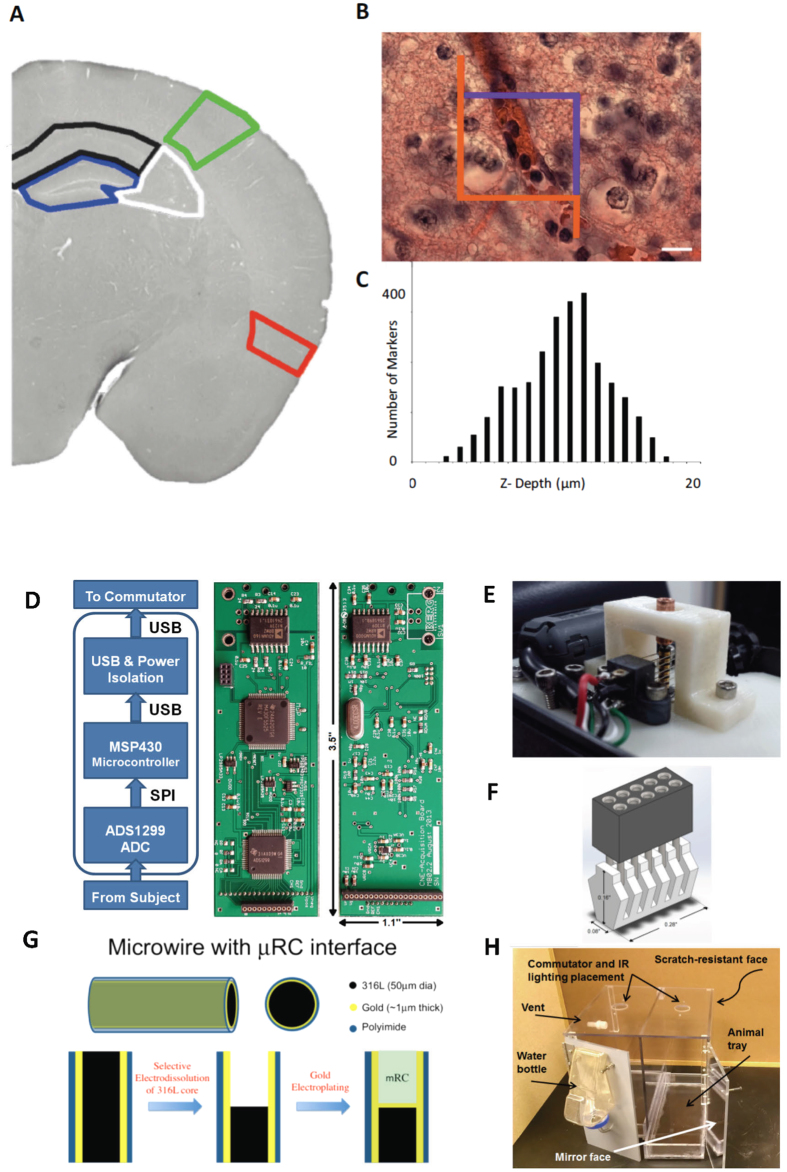
Methodology. (**A–C**) Stereological Cell Counting Methods. (**A**) Brain regions of interest analyzed including the hippocampal sub-regions of the dentate gyrus (DG) outlined in blue, the CA3 outlined in white, and the CA1 outlined in black; somatosensory cortex outlined in green; and entorhinal cortex outlined in red. In **B** is shown a hematoxylin and eosin stained specimen with a superimposed optical fractionator counting frame (45 μm × 45 μm). Cells touching the purple boundary or inside the box are counted, but not if they touch an orange line. In **C** is shown a representative histogram demonstrating the number of cells counted along each plane of a single microscope slide. The top of the slide represents the first plane to come into focus. Note that a 2 μm guard zone was placed at the top and bottom of the 18 μm optical dissector depth. Scale bar for image A is 1000 μm and for image B is 15 μm. (**D–H**) Recording System Components. (**D**) Functional Schematic and photographs of custom 8-channel recording amplifier that includes a 24-bit digitizing analog front end, a microcontroller and power conditioning middle, and electrical isolation for power and USB. (**E**) Custom low-torque 4-circuit commutator that allows the recording amplifier to hang below it and permit free rotational motion of the cabled animal. (**F**) Custom headmount connector. (**G**) Micro-reaction chamber (μRC) electrodes created from hollowed out 50 μm gold coated stainless steel (type) wires internally deposited with iridium oxide to create very low electrical impedance electrodes. These μRC electrodes have reduced recording noise, and maintain quality recordings over the long periods of time required to perform these chronic experiments. (**H**) Custom designed animal housing cages that permit long-term video and electronic recordings from implanted animals.

An optical fractionator method was used to estimate the total number of cells (Stereology Investigator, MicroBrightField, Inc., used in conjunction with an Olympus BX51WIF microscope with 3-axis motor controlled stage, Mac 5000, LUDL Electronics products, LTD.). A three-dimensional optical dissector counting probe (x, y, z dimension of 100 × 75 × 21 μm, respectively) was applied to a systematic random sample of sites (mean 75, sd 24) in each region (Fig. 6A).

Cells were counted using a 100x oil immersion objective lens (numerical aperture = 1.4) with a mean section thickness of 17.3 μm (sd 1.7) and a guard zone of 2 μm at the top and bottom of each slide (Fig. 6C). A dissector height of 18 μm, a 45 × 45 μm counting frame (Fig. 6B) and a sampling grid area (xy) that covered the region of interest (Fig. 6A). The tissue shrinkage was corrected on every section and at every site before counting the cells in the region of interest (ROI). The cell nucleus top was used as the fiducial point for WBC and neurons, while the widest cell diameter was used for RBC and parasites. Cells touching the purple boundary or between the purple and orange line but not touching the orange line of the counting frame (Fig. 6B) are included in the count, classified by size and morphology as WBC, RBC, iRBC, or neuron, and digitally marked.

#### Cell Count Per Region

The stereological estimate of total number of cells (N) of each type is derived from the number of cells of each type counted (WBC, iRBC, and RBC) divided by the volume fraction region sampled.

The total number (*N*) of WBC, iRBC, RBC and neurons in each region were estimated by the following formula:


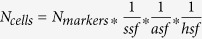


where *ssf* is section-sampling fraction (number of sections analyzed/number of sections including the region of interest), *asf* is area sampling fraction (area of counting frame/area of sampling grid) and *hsf* is the height sampling fraction (optical dissector height/average slice thickness).

The optical fractionator variables were adjusted such that the Gundersen coefficient of error (CE) for WBC counts was below 0.1 for a pilot sampling of slides across cohorts^33^. In most cases, other cell counts were higher than WBC, and therefore the CE levels lower. In practice, this meant that a typical counting frame has at least one WBC^28^.

#### Cavalieri Volume Estimate for each Region

The Cavalieri estimator probe was used to estimate the total volume in each counted ROI. A random sampling grid sized 50 × 50 μm was laid over each ROI and the total volume was calculated (using a shape factor of 4). Coefficients of error^33^ (m = 1) for volume estimates were less than 0.05.

#### Cell Density Estimates

The cell density was calculated by dividing the total cells in each ROI by the volume. The total hippocampal cell density was obtained by summing the total cell counts of the hippocampal subregions and dividing by the sum of the subregion volumes.

#### Statistical Comparisons of Cell Densities

Cell densities (RBC, iRBC, WBC) averages were computed within cohort and normalized to the mean cell density in the mouse-strain specific control group. WBC to RBC ratios were computed on an animal-wise basis and cohort means and standard deviations computed. Error bars represent the standard errors of the means of these ratios.

### Chronic Monitoring of Treated Animals

Cohorts of animals were inoculated at P23 and then treated with Artesunate. They were then implanted with electrodes and monitored long term for clinical signs of epilepsy using continuous 24/7 video and electrophysiological monitoring. Physiological biopotentials were collected from hippocampal depth, cortical screw, and neck electromyogram (EMG) electrodes. In some animals, a lead was embedded in the precordium to collect electrocardiogram (EKG) potentials.

#### Electrode Details

Hippocampal depth electrodes were fabricated from 50 μm gold-plated 316 L stainless steel wire insulated with polyimide (California Fine Wire) formed with micro-reaction chamber (μRC) ends^32^ to provide ultra-low impedance (typically <3 kΩ) and excellent DC sensitivity. The stainless steel core is etched out at the end to form a chamber, the inside of which is electro-deposited with gold and iridium oxide (Fig. 6G) to form a high-surface area high-charge passing capacity electrochemical interface without increasing the geometric surface area of the interface with brain.

Cortical activity was monitored from stainless steel screw electrodes (#000 self-tapping, Morris Co.). Neck muscle activity was monitored from 3 mm exposed lengths of 50 μm diameter gold-plated 316 L stainless steel wire insulated with polyimide. Electrode ends were prepared by dissolving the polyimide in heated nano-strip solution (KMG Inc.), and electroplating with gold to avoid bimetallic corrosion reactions. Precordium EKG leads were formed from same 50 μm gold-plated 316 L stainless steel wire, with the exposed end formed into a ~2 mm diameter coil and backed with a thin layer of biocompatible polymer (Sylgard 184).

#### Head-mounted Connector Details

The head mount (Fig. 6F) was made of two pieces: a 5 × 2, 50 mil-pitch female connector (Mill-Max Mfg. Corp.), and a 3D printed multi-grooved mating piece that receives the connector pins on one side and the electrodes on the other. The lower piece has a narrow base to sit on the surface of the skull between the electrodes. The head-mount has mass of 0.34 g. For ease of implant, leads that can be are soldered to the connector pins prior to implant. The others connected using a conductive silver paste (PELCO Colloidal Silver Paste, Ted Pella Inc.) during the surgical procedure. The grooves in the headmount help to prevent in this procedure shorting to nearby channels.

#### Stereotaxic surgeries

Animals were implanted after a minimum of 5 days, when parasitemia was consistently zero, and typically 20 days post inoculation. Surgery was done under inhalational anesthesia (isoflurane), application of local anesthetic (bupivacaine) and followed by long-lasting analgesic (Ketoprofen). Epidural stainless steel screw electrodes were placed over frontal cortex (AP+1.5, ML ± 2.5 mm) and over S1 cortex (AP −2.3, ML ±3.5 mm) for anterior and posterior cortical measurement, and at AP −3.8, ML ±3.5 mm for reference and ground respectively. Bilateral μRC depth electrodes were placed into hippocampus (AP −2.3, ML ±2.0, DV −1.5 mm) and secured with cyanoacrylate glue. All measurements are bregma-referenced.

The headmount was secured to the to the skull with cyanoacrylate glue. Neck EMG electrodes that were pre-soldered to the headmount were placed into a bluntly dissected opening within in the nuchal muscles and secured with polyglycolic acid absorbable sutures through the subcutaneous tissue. PFA-insulated gold wires (AM Systems) that were pre-soldered to headmount connector were then wrapped around the screw heads and electrically connected with silver paste. EKG electrodes were implanted through a small incision in the chest, placed in a bluntly dissected hole in the precordium and sutured into place, and the lead subcutaneously routed to the headmount and electrically connected with silver paint. After electrical connections were made the construct was embedded in dental cement. After surgery, animals were moved to individual cages and given moistened food, and health was monitored for a minimum of 7 days, including daily weights, fur grooming behavior and feeding. Animals were allowed to recover for at least one (but typically 3–5) days before recordings were initiated.

#### Video-EEG Recording System

Following implant, animals were housed individually in custom cages designed for recording. Cages are made out of polycarbonate and are designed to each contain two separate 6″ × 12″ enclosures (Fig. 6H). The front face and back wall of the cages are scratch resistant and mirrored polycarbonate material, respectively. The base contains a removable bedding tray to allow changing and cleaning without having to disrupt the recording.

The data acquisition board hangs down through a 1.5 inch hole in the ceiling of the cage from a commutator fastened to the cage top. Infrared lighting, water bottle holders, and appropriate ventilation are also incorporated.

Recordings were collected for each animal continuously (i.e. twenty-four hours, seven days a week) for extended periods for the duration of their lifetime. For most animals, these recordings were uninterrupted except to satisfy brief equipment maintenance requirements. In some cases, especially associated with control animals, recordings were interrupted after periods greater than 3 weeks to allow monitoring of other animals.

#### Commutator

In order to ensure the mouse’s freedom of movement inside the cage a commutator was built to allow for rotational movement of the acquisition board as the mouse moves inside the cage. To pass USB signals, four-circuit commutators were custom fabricated from miniature slip ring assemblies (AC2690 and AC259, Moog, Inc.) along with ultra-low torque ball bearings, 3D printed supporting structures and a commercially available housing (Bud Industries). Ferrite core filters (TDK Corporation) are included on either end of the USB cable to minimize electromagnetic interference.

#### Acquisition Board

A custom data acquisition board was designed to provide 8 channels of very high fidelity biopotential recording at a low cost and very low size and power. Because the digitizing amplifier hangs from the commutator, it minimizes strain on the animal while providing high fidelity digitized signals, and minimizes the number of commutator circuits required.

The data acquisition board consists of three main subsystems: the analog front end (AFE), a microcontroller, and the data and power isolation, as illustrated in Fig. 6D.

The AFE subsystem is a commercially available low-noise 8-channel bio-potential amplifier (ADS1299, Texas Instruments) that for each channel provides a programmable gain amplifier and 24-bit delta-sigma analog to digital converter (∆Σ-ADC). The inherent oversampling of the noise rejection of the ∆Σ-ADC combined with the architectural design of our recording system and low impedance of the electrodes provides high signal quality with minimal environmental 60 Hz noise without a head-mounted preamplifier. The 24-bit digitization provides dynamic range of 4.5 V with sub-microvolt divisions, and therefore we use no AC filtering prior to digitization. With an internal gain of 8 and acquisition rate of 1000 samples/second per channel, as used for most of the included recordings, our measured input-referred noise for these boards is ~1 μV, and common mode rejection ratio at 10 Hz of about −100 dB. The microcontroller pulls the digitized signals from the AFE and communicates with the computer through USB, to which it appears as a virtual communication port. Custom software written with Labview (National Instruments) is used to view the data in real-time and write it to disk. The whole amplifier is powered from the USB bus.

#### Seizure Scoring and Classification

Recorded signals were hand scored using an in-house data pager written in Labview that allows for on-line variable re-referencing, filtering, spectral analysis and annotation.

Spontaneous seizures of more than 10 seconds duration were identified and scored for origin and evolution. Electrographic origins were characterized as: subcortical, cortical, or unknown origin within the limitations of the recording montage. Evolution was characterized as: focal seizure, secondary generalization, or unknown generalization. Seizures that appeared simultaneously from multiple brain regions were classified with unknown origin and evolution. Behavioral correlates were then identified from video.

For each seizure, behavior was classified using a modified Racine scale^30^. The scale was as follows: 0, no behavioral alteration; 1, head nodding; 2, automatisms, tail stiffening and wet dog shakes; 3, previous symptoms plus unilateral forelimb clonus; 4, addition of bilateral forelimb clonus and rearing; 5, continuous rearing and falling; and 6, violent jumping, rearing and falling.

### Chronic Monitoring Quantification and Statistical Analysis

#### Survival Analysis

All animals inoculated for chronic recording under the 7 studied cohorts (4 infected, 3 control) were included in the survival analysis. Animals were censored if they died from surgical implant or were sacrificed within protocol. Statistical significance of differences between observed hazard functions were estimated with a two-sample log-rank test^31^ over the periods 0–28 and 0–45 days.

#### Inclusion Criteria for determination of epilepsy

All recorded data was reviewed for and marked for electrographic seizures. Animals were included in further analysis (and reported in Fig. 2) if they survived at least to 26 days post inoculation, if we collected at least 2 days of recording, and they did not suffer a malarial recrudescence. Seizures were counted for purposes of Figs 2 and 4 if they were at least 10 seconds long. Animals were scored as epileptic if: (1) they had at least 2 seizures, (2) seizures at least 3 days past implant, and (3) at least one seizure more than 26 days post inoculation.

#### Epilepsy Rates

Epilepsy rates in Table 1 were estimated using a plug-in estimator of the number observed divided by the reference number (initial cohort size or number at day 26), and uncertainties were estimated from binomial statistics. Significance values were computed from a conservative upper bound estimated epilepsy rate for uninfected animals estimated as 1/(N_controls_ + 1), where N_controls_ is the ensemble number of control animals. Reported p values are therefore the probability of observing the experimental numbers if the actual epilepsy rate was 1/22.

## Additional Information

**How to cite this article:** Ssentongo, P. *et al*. A Murine Model to Study Epilepsy and SUDEP Induced by Malaria Infection. *Sci. Rep.* **7**, 43652; doi: 10.1038/srep43652 (2017).

**Publisher's note:** Springer Nature remains neutral with regard to jurisdictional claims in published maps and institutional affiliations.

## Supplementary Material

Supplementary Information

## Figures and Tables

**Table 1:** Chronic Recording Statistics.

Cohort	Number Inoculated (Day 0)	Mortality Before Day 21	Number Chronically Recorded	Number Recorded With Epilepsy	Epilepsy Rate (of inoculated)	Epilepsy Rate (of recorded)	Seizure Latency days post inoculation Median (Range)	Mortality After Day 26	Seizure Associated Mortality	SUDEP
SW-PbNK65	25	4	17	13	0.52 ± 0.10 (p = 1e-11)	0.76 ± 0.10 (p = 7e-15)	39 (29–139)	7	7	2
SW-PbANKA	34	23	10	7	0.21 ± 0.07 (p = 6e-4)	0.70 ± 0.14 (p = 4e-8)	30 (22–105)	5*	3	1
C57BL/6-PbANKA	26	12	12	9	0.35 ± 0.09 (p = 2e-6)	0.75 ± 0.12 (p = 2e-10)	47 (38–95)	1	0	0
CBA-PbANKA	5	1	4	3	0.60 ± 0.22 (p = 9e-4)	0.75 ± 0.22 (p = 3e-4)	72 (54–116)	1	0	0
SW-Control	9	0	9	0	0 (p = 0.7)	0 (p = 0.7)		0	0	0
C57BL/6-Control	7	0	7	0	0 (p = 0.7)	0 (p = 0.7)		0	0	0
CBA-Control	5	0	5	0	0 (p = 0.8)	0 (p = 0.8)		0	0	0

Statistics from seven cohorts of mouse-strain and parasite/control combinations studied chronically for development of epilepsy from CM. Animals were inoculated at P23 (denoted day 0 here) with infected or uninfected donor blood, then treated with artesenate starting at the peak of cerebral malaria. At time points well past recovery, animals were implanted with electrodes and monitored with continuous video/iEEG recordings. Infected cohorts had between 16–67% mortality rates during the acute infection and recovery time defined up to Day 21, with the highest mortality observed in the SW-PbANKA strain. Listed are the counts of animals recorded that met inclusion criteria to diagnose epilepsy (recorded at least 2 days, alive past day 26, no malaria recrudescence), the number of those that became epileptic (at least 2 seizures longer than 10 s, at least one seizure past day 26), and estimated epileptic rates (±SD estimated from binomial statistics) as fraction of inoculated and as fraction of those surviving to recording. Seizures were not observed, and therefore no epilepsy, among any of the 21 control animals across all three mouse strains. P-values for epilepsy rates were estimated from a null-hypothesis of a rate of 1 epileptic control animal in 22 (N_controls_ + 1) as function of inoculated and recorded numbers. Mortality past day 26 excludes surgical deaths, but includes 2 late malarial recrudescences (denoted with *). Seizure related mortality details counts of spontaneous deaths in seizure animals; SUDEP counts detail animals who died within 2 hours of an electrographic seizure. No early or late mortality was observed in the controls.
