# Supplementary material for: A Murine Model to Study Epilepsy and SUDEP Induced by Malaria Infection
Source: Sci Rep. 2017 Mar 8;7:43652. doi: 10.1038/srep43652 (PMC5341121; doi:10.1038/srep43652)
Supplement: Supplementary Information [file srep43652-s1.doc]

**Supplementary Material**

**A Murine Model to Study Epilepsy and SUDEP Induced by Malaria Infection**

Paddy Ssentongo^a,b,c^, Anna E. Robuccio^a^, Godfrey Thuku^a,b^, Derek G. Sim^d,e^ , Ali Nabi^a,b^, Fatemeh Bahari^a,b^, Balaji Shanmugasundaram^a,b^, Myles W. Billard^a,b^, Andrew Geronimo^a,b,f,g^, Kurt W. Short^a,b,f^, Patrick J. Drew^a,b,f^, Jennifer Baccon^f,h^, Steven L. Weinstein^i^, Frank G. Gilliam^f,g^ , José A. Stoute^l^, Vernon M. Chinchilli^c^, Andrew F. Read^d,e^, Bruce J. Gluckman^a,b,f,j,+^, Steven J. Schiff^a,b,d,f,k,+,*^

^+^ Contributed Equally

1. Center for Neural Engineering, Penn State University, University Park, Pennsylvania 16802, USA
2. Department of Engineering Science and Mechanics, Penn State University, University Park, Pennsylvania 16802, USA
3. Department of Public Health Sciences, Penn State College of Medicine, Hershey, Pennsylvania 17033, USA
4. Center for Infectious Disease Dynamics, Penn State University, University Park, Pennsylvania 16802, USA
5. Departments of Biology and Entomology, Penn State University, University Park, Pennsylvania 16802, USA
6. Department of Neurosurgery, Penn State College of Medicine, Hershey, Pennsylvania 17033, USA
7. Department of Neurology, Penn State College of Medicine, Hershey, Hershey, Pennsylvania 17033, USA
8. Department of Pathology, Penn State College of Medicine, Hershey, Hershey, Pennsylvania 17033, USA
9. Department of Neurology, Children's National Medical Center, George Washington University, Washington, DC 20010, USA
10. Department of Bioengineering, Penn State University, University Park, Hershey, Pennsylvania, 16803, USA
11. Department of Physics, Penn State University, University Park, Pennsylvania, 16803, USA
12. Department of Medicine, Penn State University College of Medicine, Hershey, Pennsylvania 17033, USA

Re-submitted to Scientific Reports, December 22, 2016

**Supplementary Material**

| **Cohort type** | **Brain Region** | **WBC: RBC/**  **Geometric mean** | **WBC: RBC/ lower and upper 95%Confidence limits** | | **WBC: RBC**  **Normalized to controls/Geometric mean** | **WBC: RBC**  **Normalized to controls/ lower and upper 95%Confidence limits** | |
| --- | --- | --- | --- | --- | --- | --- | --- |
| SW-PbNK65 | EC | **0.80** | **0.54** | **1.21** | 3.36 | 2.08 | 5.44 |
|  | Hip | 0.08 | 0.07 | 0.10 | 0.50 | 0.037 | 0.67 |
|  | S1 | 0.27 | 0.23 | 0.31 | 2.43 | 1.93 | 3.07 |
| SW-Pb ANKA | EC | 0.53 | 0.40 | 0.70 | 2.21 | 1.51 | 3.23 |
|  | Hip | 0.48 | 0.40 | 0.55 | 2.82 | 2.18 | 3.65 |
|  | S1 | 0.33 | 0.11 | 0.34 | 1.79 | 0.99 | 3.23 |
| SW-CONTROL | EC | 0.24 | 0.19 | 0.31 | N/A | N/A | N/A |
|  | Hip | 0.17 | 0.14 | 0.21 | N/A | N/A | N/A |
|  | S1 | 0.14 | 0.09 | 0.13 | N/A | N/A | N/A |
| C57BL/6-PbNK65 | EC | 0.58 | 0.49 | 0.68 | 20.33 | 11.23 | 36.80 |
|  | Hip | **1.42** | **1.16** | **1.62** | 53.33 | 34.67 | 82.01 |
|  | S1 | **0.98** | **0.89** | **1.06** | 18.17 | 11.67 | 28.29 |
| C57BL/6-PbANKA | EC | 0.29 | 0.24 | 0.36 | 10.06 | 4.89 | 20.67 |
|  | Hip | 0.45 | 0.40 | 0.52 | 17.65 | 11.62 | 26.80 |
|  | S1 | 0.19 | 0.18 | 0.21 | 3.62 | 2.33 | 5.61 |
| C57BL/6-CONTROL | EC | 0.04 | .02 | .06 | N/A | N/A | N/A |
|  | Hip | 0.03 | 0.02 | 0.04 | N/A | N/A | N/A |
|  | S1 | 0.53 | 0.03 | 0.08 | N/A | N/A | N/A |
| CBA-PbNK65 | EC | **1.44** | **1.18** | **1.66** | 20.33 | 11.23 | 36.80 |
|  | Hip | **1.50** | **1.35** | **1.65** | 68.79 | 59.11 | 80.04 |
|  | S1 | **0.94** | **0.81** | **1.08** | 9.07 | 5.87 | 13.99 |
| CBA-Pb ANKA | EC | 0.04 | 0.04 | 0.12 | 5.19 | 2.89 | 9.32 |
|  | Hip | 0.30 | 0.24 | 0.36 | 13.66 | 10.83 | 17.23 |
|  | S1 | 0.29 | 0.24 | 0.34 | 2.77 | 1.78 | 4.33 |
| CBA-CONTROL | EC | 0.07 | 0.04 | 0.12 | N/A | N/A | N/A |
|  | Hip | 0.02 | 0.02 | 0.02 | N/A | N/A | N/A |
|  | S1 | 0.11 | 0.09 | 0.13 | N/A | N/A | N/A |

**Table S1**. Geometric mean ratios of white and red blood cells (WBC: RBC) in the infected mice with their 95% confidence intervals (generalized linear model). Several regions in different strain combinations revealed WBC: RBC ratios whose upper bound confidence limits exceeded 1.0, indicating that there were more WBCs than RBCs within the microvasculature. To our knowledge, such WBC:RBC ratios have not been reported in human CM, and we used this as the basis for our decision to exclude strain combinations C57BL/6-PbNK65 and CBA-PbNK65 from chronic studies of postmalarial epilepsy. There were ten (N=10) mice in each strain combination cohort within the table. Values in bold indicate that confidence limits exceeded the ratio 1.0. These values are consistent with ratios and confidence limits when normalized by strain specific controls.
